# Global warming without global mean precipitation increase?

**DOI:** 10.1126/sciadv.1501572

**Published:** 2016-06-24

**Authors:** Marc Salzmann

**Affiliations:** Institute for Meteorology, Universität Leipzig, Vor dem Hospitaltore 1, 04103 Leipzig, Germany. Email: marc.salzmann@uni-leipzig.de

**Keywords:** Climate modeling, global warming, global mean precipitation, hydrological sensitivity, aerosol, CMIP5, anthropogenic climate change

## Abstract

Global climate models simulate a robust increase of global mean precipitation of about 1.5 to 2% per kelvin surface warming in response to greenhouse gas (GHG) forcing. Here, it is shown that the sensitivity to aerosol cooling is robust as well, albeit roughly twice as large. This larger sensitivity is consistent with energy budget arguments. At the same time, it is still considerably lower than the 6.5 to 7% K^−1^ decrease of the water vapor concentration with cooling from anthropogenic aerosol because the water vapor radiative feedback lowers the hydrological sensitivity to anthropogenic forcings. When GHG and aerosol forcings are combined, the climate models with a realistic 20th century warming indicate that the global mean precipitation increase due to GHG warming has, until recently, been completely masked by aerosol drying. This explains the apparent lack of sensitivity of the global mean precipitation to the net global warming recently found in observations. As the importance of GHG warming increases in the future, a clear signal will emerge.

## Teaser

Robust response of the hydrological cycle to aerosol has thus far suppressed global mean precipitation increase.

## INTRODUCTION

Climate model simulations suggest that the global mean precipitation will increase by 1.5 to 2% K^−1^ surface warming in response to greenhouse gas (GHG) forcing (*1*, *2*). However, this expected increase is not yet generally supported by observations (*3*, *4*), and it has recently been suggested that the hydrological sensitivity might be lower than expected because of a cloud radiative feedback that is not represented by climate models (*5*). Meanwhile, an issue that has received little attention is the hydrological sensitivity associated with an increase in anthropogenic aerosol. On the one hand, it is well understood that the effect of anthropogenic aerosol is net cooling and drying, that aerosol cooling has reduced the overall anthropogenic warming (*6*–*8*), and that a reduction in solar radiation yields a stronger hydrological response than GHG warming (*9*, *10*). Various studies (*11*–*14*) have shown that aerosol has to be taken into account for explaining observed precipitation trends and that it is important for determining the overall hydrological sensitivity. On the other hand, it is still very often implicitly assumed that the global mean hydrological sensitivity to aerosol cooling is the same as that to GHG warming. However, the hydrological sensitivity to GHG forcing is lowered by the long-wave (infrared) radiative effect of GHGs, which tends to prevent condensation heat from escaping to space: whereas the water vapor concentration in the boundary layer increases by about 6.5 to 7% K^−1^ surface warming, precipitation increases only by 1.5 to 2% K^−1^ surface warming for GHG forcing (*1*, *2*, *12*, *15*, *16*). Anthropogenic aerosols, such as sulfates that primarily scatter sunlight, on the other hand, have a comparatively small long-wave radiative effect. They therefore exhibit larger precipitation sensitivity and a weaker damping effect (although absorbing and scattering aerosols can also induce damping or compounding effects on precipitation sensitivity).

## RESULTS

The “historicalGHG” model sensitivity experiment from the Coupled Model Intercomparison Project Phase 5 (CMIP5) (*17*) yields a multimodel mean sensitivity of 1.7 ± 0.4% K^−1^ (mean ± 1 SD) to well-mixed GHGs, as expected. The multimodel mean sensitivity to aerosol forcing, on the other hand, is roughly twice as large and also rather robust across different models (3.6 ± 0.5% K^−1^, based on eight CMIP5 models for which aerosol-only runs are available; see the Supplementary Materials for details). However, this sensitivity to aerosol cooling is still significantly lower than the 6.5 to 7% K^−1^ change of the lower tropospheric water vapor content, which is simulated in global climate models in response to surface temperature changes and which coincides with the expectations based on the Clausius-Clapeyron relation, under the assumption of constant relative humidity (*2*). Instead, it is similar to the sensitivity that is obtained by comparing two CMIP5 experiments with fixed sea surface temperatures (SSTs), in one of which the SST is increased by 4 K everywhere (see table S1 and fig. S1) at constant forcing, allowing only atmospheric feedbacks. This suggests a significant contribution from the water vapor long-wave radiative feedback and from long-wave cloud feedbacks to the overall damping, in agreement with previous studies (*12*, *13*). The magnitude of this damping (roughly half the GHG damping) is compatible with the contribution of the water vapor feedback to the overall increased greenhouse effect [roughly a doubling (*18*)].

**Fig. 1:**
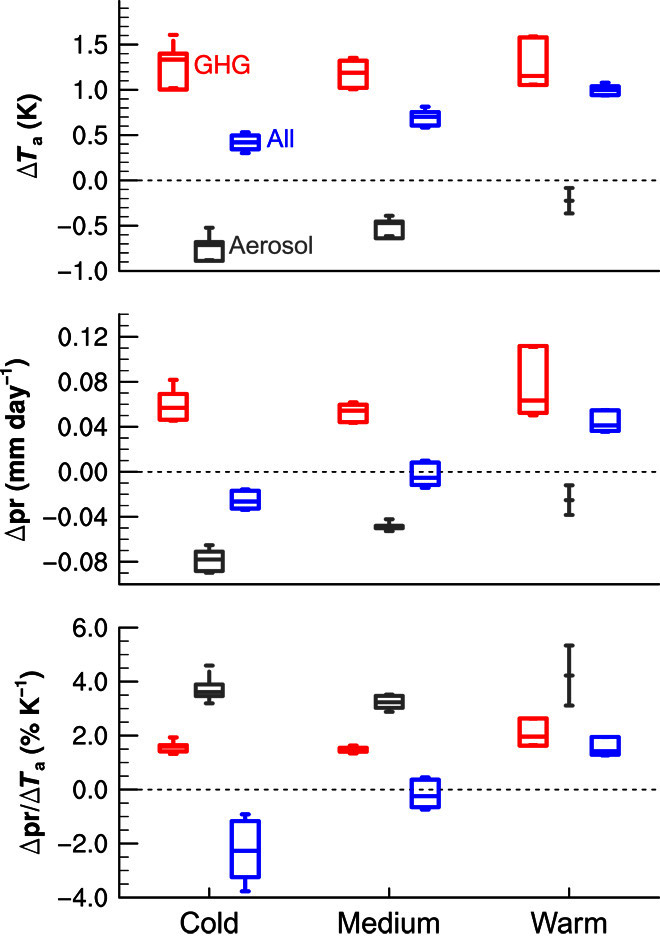
Response to GHG, aerosol, and all forcings. Multimodel mean difference between years 1850–1869 and 1986–2005 from climate model runs with only GHG (red), only aerosol (gray), and all forcings (blue) for global mean near-surface air temperature (**top**), precipitation (**middle**), and hydrological sensitivity (**bottom**). The models are grouped into cold, medium, and warm models based on 20th century warming in the historical (all-forcing) runs according to fig. S2. Boxes indicate medians and quartiles. The ranges indicate averages ± 1 SD.

Here, the hydrological sensitivities have been estimated from differences in surface air temperature Δ*T* and precipitation Δ*P* between the years 1850–1869 and 1986–2005 from climate model runs with only GHG, only aerosol, and all forcings (Fig. 1), and the models have been grouped according to the magnitude of their 20th century warming in the all-forcing runs (fig. S2). Because the natural forcing is comparatively small, Δ*T* and Δ*P* in the all-forcing runs are given by the sums of Δ*T* and Δ*P* from the GHG and aerosol runs, that is, Δ*T* = Δ*T*_G_ + Δ*T*_A_ and Δ*P* = Δ*P*_G_ + Δ*P*_A_ to a very good approximation (fig. S3). Consequently, the overall hydrological sensitivity can be computed from

$$\frac{\delta P}{\delta T}=\frac{\Delta P_{G}+\Delta P_{A}}{\Delta T_{G}+\Delta T_{A}}$$ (1)

which yields a rather accurate estimate according to fig. S3. Therefore, the global mean precipitation change is related to the individual hydrological sensitivities as follows

$$\Delta P=\frac{\delta P}{\delta T}\Delta T={\left(\frac{\delta P}{\delta T}\right)}_{G}\Delta T_{G}+{\left(\frac{\delta P}{\delta T}\right)}_{A}\Delta T_{A}$$ (2)

where ${\left(\frac{\delta P}{\delta T}\right)}_{G}$ and ${\left(\frac{\delta P}{\delta T}\right)}_{A}$ are the hydrological sensitivities from the single-forcing experiments. Because Δ*T*_G_ > 0 and Δ*T*_A_ < 0 and also ${\left(\frac{\delta P}{\delta T}\right)}_{A}>{\left(\frac{\delta P}{\delta T}\right)}_{G}$, the actual (all-forcing) hydrological sensitivity is lower than the known and often discussed sensitivity to GHGs (compare schematic in fig. S4). Overall, the simulated multimodel mean hydrological sensitivity is −0.4 ± 1.7% K^−1^ in the standard historical experiment that combines all forcings.

Figure 1 together with fig. S2 shows that the models that simulate a fairly realistic 20th century warming (“medium”) tend to yield particularly small overall hydrological sensitivities, although it must be noted that on average, the medium models slightly underestimate the observed warming, whereas the “warm” models yield several individual runs with only a rather small overestimate of the global mean temperature increase. This suggests that the overall hydrological sensitivity is still much smaller than the hydrological sensitivity to GHGs and also still within the range of internal climate variability given by the spread between individual model runs in fig. S3. It also explains the absence of a strong hydrological sensitivity in observations (*4*) and suggests that global mean precipitation has not yet increased significantly despite global warming simply because the hydrological sensitivity to aerosol cooling is larger than that to GHG warming. This lack of observed response in global precipitation to GHG warming is consistent with energy budget arguments and the analysis of historical trends in previous studies that have taken into account aerosol effects (*14*, *16*).

**Fig. 2:**
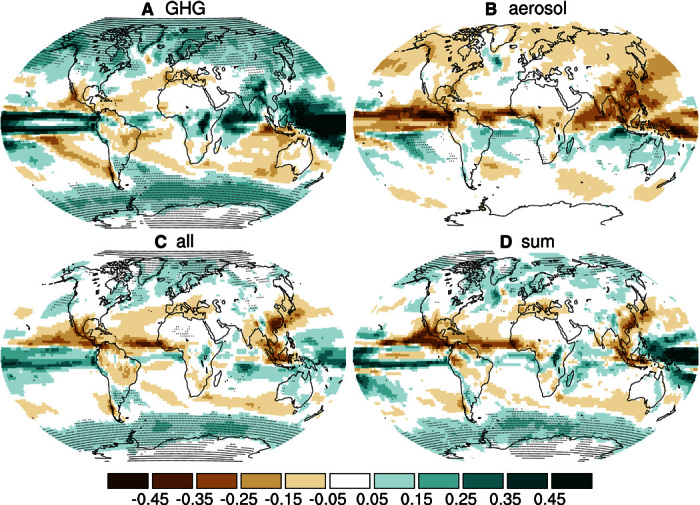
Regional precipitation response and additivity of responses to individual forcings. Multimodel averages of simulated surface precipitation change between years 1850–1869 and 1986–2005 in millimeters per day for GHG, aerosol, and all forcings, as well as the sum of the GHG- and aerosol-forcing experiments for models for which at least one aerosol run is available. Stippling indicates that six of seven models (where two very similar models have been considered as a single model) agree on the sign of the change.

However, locally, the changes due to GHGs and aerosol do not balance (Fig. 2 and figs. S5 to S7) because the aerosol forcing is highly nonuniform (*19*), which makes the detection of anthropogenic changes possible (*20*). Yet, to completely understand observed regional patterns of multidecadal precipitation trends, one has to take into account not only anthropogenic forcings but also internal climate variability (*21*–*23*). Although anthropogenic aerosol can have large influences on local circulation (*24*, *25*), the overall effect on the global mean atmospheric vertical overturning circulation strength is much weaker than that of GHGs (figs. S8 and S9). This is in line with the weaker damping and higher hydrological sensitivity, because the low sensitivity to GHG warming is associated with a weakening of the circulation under GHG warming (*2*). Ultimately, the local response of precipitation to anthropogenic forcing is determined not only by the temperature-dependent water vapor availability and the strengthening or weakening of the overturning circulation but also by geographical shifts of precipitation patterns (*16*). The impacts depend strongly on changes of precipitation intensity (*26*, *27*) and seasonal cycle (*28*). Furthermore, the type of aerosol is important (*11*, *16*, *29*, *30*), and also, the treatment of aerosol effects differs in global models (table S2). This strongly influences the simulated changes in temperature and precipitation. At the same time, the aerosol hydrological sensitivity is found to be fairly robust (Fig. 1). Many features of the spatial patterns simulated in response to anthropogenic aerosol (Fig. 2B) are fairly robust as well (*31*), even across different models (figs. S5 to S7).

**Fig. 3:**
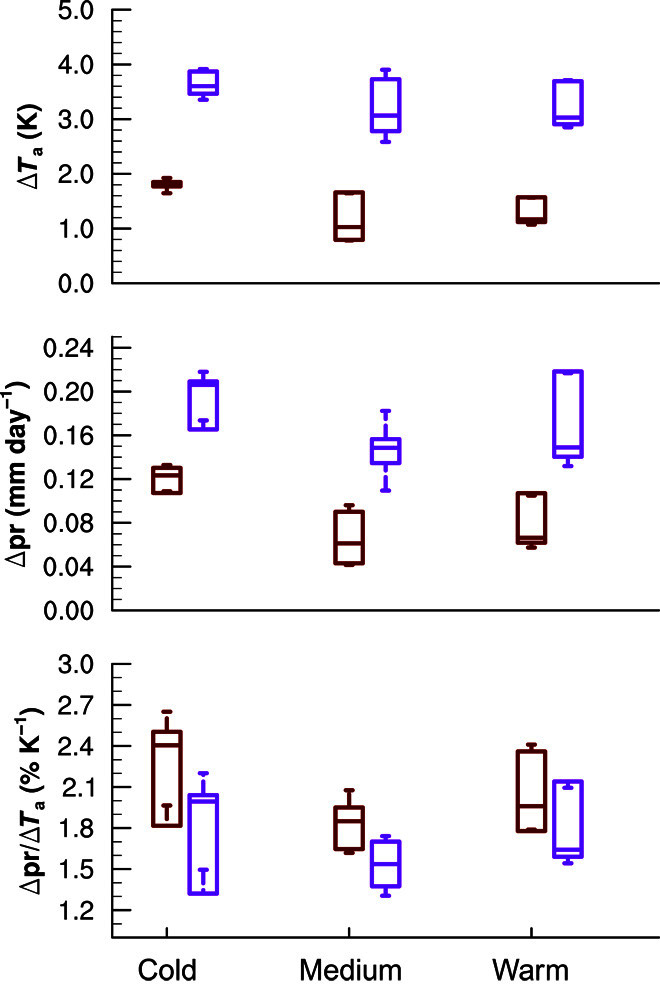
Future projections. Similar to Fig. 1 but showing differences between years 2006–2025 and 2081–2100 based on the rcp45 (brown) and the rcp85 (purple) CMIP5 future emission scenarios.

Because long-lived GHGs accumulate in the atmosphere while the atmospheric residence time of tropospheric aerosol is rather short, and because aerosol emissions are expected to decrease in the future, eventually Δ*T*_G_ will overwhelm Δ*T*_A_ (*32*, *33*), and thus the overall hydrological sensitivity will be dominated by GHGs. This is confirmed by analyzing results from CMIP5 future scenario runs in Fig. 3.

## DISCUSSION

The finding that the global mean hydrological response to aerosol is robust across the models and independent of the exact treatment and strength of the aerosol effect allows us to better understand simulated and observed changes of the global mean hydrological cycle. Because the hydrological sensitivity in the CMIP5 historical coupled model runs depends on the surface warming, the simulated hydrological sensitivities can be constrained by surface temperature observations. This constrained model-based estimate supports state-of-the-art observational estimates (*4*), showing no evidence of a discernible historical trend. Furthermore, on the basis of the arguments above, one can see that if climate geoengineering were used to reduce the global mean temperature via solar radiation management, the global mean precipitation in the resulting state would be reduced compared to what it would be at the same temperature without the added GHGs, as suggested by Bala *et al*. (*9*), Bala *et al*. (*10*), and Bony *et al*. (*15*). On average, the precipitation decrease would roughly correspond to the precipitation decrease that is found when increasing the GHG concentrations in atmospheric models while keeping the SSTs fixed, although the geographical pattern would differ. This precipitation decrease has already been simulated in early uncoupled model simulations (*1*, *34*). Conversely, reduced aerosol emissions help to “unmask” the precipitation increase by GHG warming (*35*). Eventually, the global mean aerosol effect on precipitation will almost completely be overwhelmed by GHG warming as expected on the basis of previous studies (*32*, *33*, *36*).

## MATERIALS AND METHODS

### Model data and analysis method

Model data were taken from the CMIP5 (*17*). In total, 282 atmosphere-ocean coupled model runs from 15 CMIP5 models and 24 atmosphere-only runs from 11 CMIP5 models were analyzed. For the coupled model runs, only runs from models that have performed at least one so-called single-forcing experiment in addition to the standard CMIP5 historical experiment were taken into account [as in the work by Salzmann *et al*. (*25*); see Table 1 and tables S2 to S4 for an overview]. In particular, the historicalGHG experiment takes into account only the forcing by well-mixed anthropogenic GHGs, whereas several runs from the CMIP5 historicalMisc collection of experiments included only the forcing by changing anthropogenic aerosol concentrations. Only natural forcings were included in the historicalNat runs, with the two main natural forcings being volcanic aerosol and solar variability. These single-forcing experiments facilitated the calculation of changes with respect to a given forcing, that is, the change of a climate variable, such as surface temperature or precipitation due to this particular forcing when all other forcings are kept constant. However, although the other forcings were kept constant, water vapor and clouds were still allowed to respond to the single forcing, so that these changes are not the same as partial derivatives. The single forcings were abbreviated “GHG” for anthropogenic GHG, “aerosol” for anthropogenic aerosol, and “nat” for natural forcings. In addition to these single-forcing runs, runs from the standard historical experiment were analyzed. This experiment took into account all the known anthropogenic GHG and aerosol, as well as natural forcings. The experiment was abbreviated “all.” The historical runs typically start in 1850 and end in 2005, and the differences between the first and the last 20 years were analyzed as in the study by Held and Soden (*2*), except when the runs were compared to observations. Then, the years 1901–1920 were compared to the years 1986–2005. Uncertainties and intermodel spread in the CMIP5 historical coupled model runs stem from uncertainty in simulated aerosol radiative forcing, cloud radiative feedback strength, and ocean heat uptake.

**Table 1:** Models. CNRM, Centre National de Recherches Météorologiques; CERFACS, Centre Européen de Recherche et de Formation Avancée en Calcul Scientifique; CSIRO, Australian Commonwealth Scientific and Industrial Research Organisation, in collaboration with the Queensland Climate Change Centre of Excellence; LASG, State Key Laboratory Numerical Modeling for atmospheric Sciences and geophysical Fluid Dynamics; IAP, Institute of Atmospheric Physics of the Chinese Academy of Sciences; CESS, Center for Earth System Science, Tshinghua University; FIO, First Institute of Oceanography, State Oceanic Administration; NOAA, National Oceanic and Atmospheric Administration; NASA, National Aeronautics and Space Administration; JAMSTEC, Japan Agency for Marine-Earth Science and Technology; AORI, Atmosphere and Ocean Research Institute, The University of Tokyo; NIES, National Institute for Environmental Studies, Ibaraki, Japan.

**Model**	**Center**	**Reference**
bcc-csm1-1	Beijing Climate Center	(*40*)
CanESM2/CanAM4	Canadian Centre for Climate Modelling and Analysis	(*41*)
CCSM4	National Center for Atmospheric Research	(*42*)
CNRM-CM5	CNRM-CM5 CNRM and CERFACS	(*43*)
CSIRO-Mk3-6-0	CSIRO Marine and Atmospheric Research	(*44*)
FGOALS-g2	LASG, IAP, CESS, and FIO	(*45*)
GFDL-CM3	NOAA Geophysical Fluid Dynamics Laboratory	(*46*)
GFDL-ESM2	NOAA Geophysical Fluid Dynamics Laboratory	(*47*)
GISS-E2-H	NASA Goddard Institute for Space Studies	(*48*)
GISS-E2-R	NASA Goddard Institute for Space Studies	(*48*)
HadGEM2-ES	Met Office Hadley Centre	(*49*)
IPSL-CM5A-LR	Institut Pierre Simon Laplace	(*50*)
MIROC-ESM	JAMSTEC, AORI, and NIES	(*51*)
MIROC-ESM-CHEM	JAMSTEC, AORI, and NIES	(*51*)
MIROC5	JAMSTEC, AORI, and NIES	(*52*)
MRI-CGCM3	Meteorological Research Institute, Tsukuba, Japan	(*53*)
MPI-ESM-LR	Max Planck Institute for Meteorology	(*54*)
NorESM1-M	Norwegian Climate Centre	(*55*)

Furthermore, two runs from two experiments with prescribed SSTs were analyzed. In these amip-style runs (where amip originally stands for Atmospheric Model Intercomparison Project), all radiative forcings were taken into account, but the SSTs could not react. Instead, observation-derived SSTs for the years 1979–2005 were prescribed in the standard amip run. In the amip4K run, the prescribed SSTs were increased by 4K everywhere, but the forcings remained identical to those in the amip base run. From the difference between the global time averages, one could then compute a hydrological sensitivity for constant forcing, allowing only feedbacks. All years of the two amip runs were taken into account. The results from amip runs should, in general, be treated with caution because at the lower boundary, energy is not conserved.

Global averages were computed from the original data, whereas for maps, the model output has been regridded to a 2° × 2° grid. The term multimodel average refers to an average in which initially all the realizations (runs with slightly different initial conditions) from a given model are averaged before averaging over the models. In the maps showing model averages, the runs from the two NASA Goddard Institute for Space Studies (GISS) models were combined into one before averaging, whereas otherwise they were considered separately. The data analysis was performed using freely available software (see Acknowledgments).

### Observational data

For model evaluation purposes, SSTs from the NOAA Extended Reconstructed Sea Surface Temperature (ERSST) version 4 (*37*) were combined with 2° × 2° regridded near-surface air temperature data from the Climatic Research Unit Time Series, version 3.22 (CRU TS3.22) data set (*38*), which is based on the work by Mitchell and Jones (*39*). While processing the data, a mask based on the maximum sea ice extent from the ERSST data set was used to mask out regions that could potentially be influenced by sea ice. This approach of masking out the maximum ice extent has been chosen to account for the fact that the CMIP5 experiments analyzed here are not deterministic with respect to internal variability [see, for example, the study by Salzmann and Cherian (*23*)].

These observation-derived surface temperatures are used to constrain hydrological sensitivity based on atmosphere-ocean coupled climate model simulations. An alternative method that provides additional insights is an energy budget analysis along the lines of previous work by Andrews *et al*. (*11*), Previdi (*12*), O’Gorman *et al*. (*13*), Wu *et al*. (*14*), and Allan *et al*. (*16*). However, unfortunately, the uncertainties associated with observational estimates of the surface energy budget that could help to constrain the coupled models’ energy budgets with observations are rather large.

## Supplementary Material

http://advances.sciencemag.org/cgi/content/full/2/6/e1501572/DC1

## SUPPLEMENTARY MATERIALS

Supplementary material for this article is available at http://advances.sciencemag.org/cgi/content/full/2/6/e1501572/DC1 Notes regarding selected figures fig. S1. Hydrological sensitivity for fixed SST. fig. S2. Grouping of models according to 20th century temperature increase. fig. S3. Response to GHG, aerosol, and all forcings from individual models. fig. S4. Schematic representation of the hydrological sensitivity to various forcings. fig. S5. Zonal mean precipitation change from individual models. fig. S6. Maps of surface precipitation change from individual models (part1). fig. S7. Maps of surface precipitation change from individual models (part2). fig. S8. Global mean atmospheric overturning circulation changes for GHG, aerosol, and all forcings. fig. S9. As fig. S8 for individual model runs. table S1. Hydrological sensitivity (% K^−1^). table S2. Treatment of indirect (cloud-aerosol) radiative effects in the historical runs. table S3. CMIP5 experiments used in this study. table S4. Number of runs per model used in this study. Reference (*56*)
