# Supplementary material for: Global warming without global mean precipitation increase?
Source: Sci Adv. 2016 Jun 24;2(6):e1501572. doi: 10.1126/sciadv.1501572 (PMC4928969; doi:10.1126/sciadv.1501572)
Supplement: http://advances.sciencemag.org/cgi/content/full/2/6/e1501572/DC1 [file supp_2_6_e1501572__index.html]

Science Advances | Science Advances

## Supplementary Materials

**This PDF file includes:**

- Notes regarding selected figures
- fig. S1. Hydrological sensitivity for fixed SST.
- fig. S2. Grouping of models according to 20th century temperature increase.
- fig. S3. Response to GHG, aerosol, and all forcings from individual models.
- fig. S4. Schematic representation of the hydrological sensitivity to various forcings.
- fig. S5. Zonal mean precipitation change from individual models.
- fig. S6. Maps of surface precipitation change from individual models (part1).
- fig. S7. Maps of surface precipitation change from individual models (part2).
- fig. S8. Global mean atmospheric overturning circulation changes for GHG, aerosol, and all forcings.
- fig. S9. As fig. S8 for individual model runs.
- table S1. Hydrological sensitivity (% K−1).
- table S2. Treatment of indirect (cloud-aerosol) radiative effects in the historical runs.
- table S3. CMIP5 experiments used in this study.
- table S4. Number of runs per model used in this study.
- Reference (*56*)

Download PDF

**Files in this Data Supplement:**

- Adobe PDF - 1501572\_SM.pdf
