# Supplementary material for: Global warming without global mean precipitation increase?
Source: Sci Adv. 2016 Jun 24;2(6):e1501572. doi: 10.1126/sciadv.1501572 (PMC4928969; doi:10.1126/sciadv.1501572)
Supplement: http://advances.sciencemag.org/cgi/content/full/2/6/e1501572/DC1 [file 1501572_SM.pdf]

Science Advances

AAAS

advances.sciencemag.org/cgi/content/full/2/6/e1501572/DC1

Supplementary Materials for

Global warming without global mean precipitation increase?

Marc Salzmann

Published 24 June 2016, *Sci. Adv.* **2**, e1501572 (2016)
DOI: 10.1126/sciadv.1501572

This PDF file includes:

- Notes regarding selected figures
- fig. S1. Hydrological sensitivity for fixed SST.
- fig. S2. Grouping of models according to 20th century temperature increase.
- fig. S3. Response to GHG, aerosol, and all forcings from individual models.
- fig. S4. Schematic representation of the hydrological sensitivity to various forcings.
- fig. S5. Zonal mean precipitation change from individual models.
- fig. S6. Maps of surface precipitation change from individual models (part1).
- fig. S7. Maps of surface precipitation change from individual models (part2).
- fig. S8. Global mean atmospheric overturning circulation changes for GHG, aerosol, and all forcings.
- fig. S9. As fig. S8 for individual model runs.
- table S1. Hydrological sensitivity (% K^{-1}).
- table S2. Treatment of indirect (cloud-aerosol) radiative effects in the historical runs.
- table S3. CMIP5 experiments used in this study.
- table S4. Number of runs per model used in this study.
- Reference (*56*)

Notes Regarding Selected Figures

Figure S2 shows that the global mean temperature and precipitation response in the "all" forcing runs almost exactly equals the sum of the global mean responses from the "GHG" and the "aerosol" single forcing experiments for all models except GFDL-CM3. The aerosol cooling in this model is in part compensated by a continual release of heat from the ocean into the atmosphere during the 20th century (*56*).

In fig. S7 the 850 hPa pressure vertical velocity ω in regions of monthly mean net ascent ($\omega < 0$) is analyzed. A considerable fraction of the precipitation in global models is, however, produced by the deep convection parameterization. Since the deep convection parameterizations computes only moistening and heating rates, the actual air mass transport is handled by the resolved dynamics. Thus, the negative of ω can be considered a (somewhat imperfect) proxy of the total air mass flux out of the boundary layer. The percentage changes in fig. S7 are similar to the percentage changes shown in (*2*), who exclusively focused on air mass fluxes diagnosed inside the parameterization. In the parameterizations, these air mass fluxes are assumed to be balanced within the same model column, so that there is no exchange of air mass between the parameterization and the resolved dynamics. The main goal of fig. S7 is to show that aerosol and GHGs differ in their effect on the overturning circulation as expected based on mass and energy balance arguments. In the absence of circulation changes and assuming constant relative humidity, one would expect the precipitation to increase by approximately by 6.5 to 7% per Kelvin surface warming based on the Clausius-Clapeyron relation (*2*).

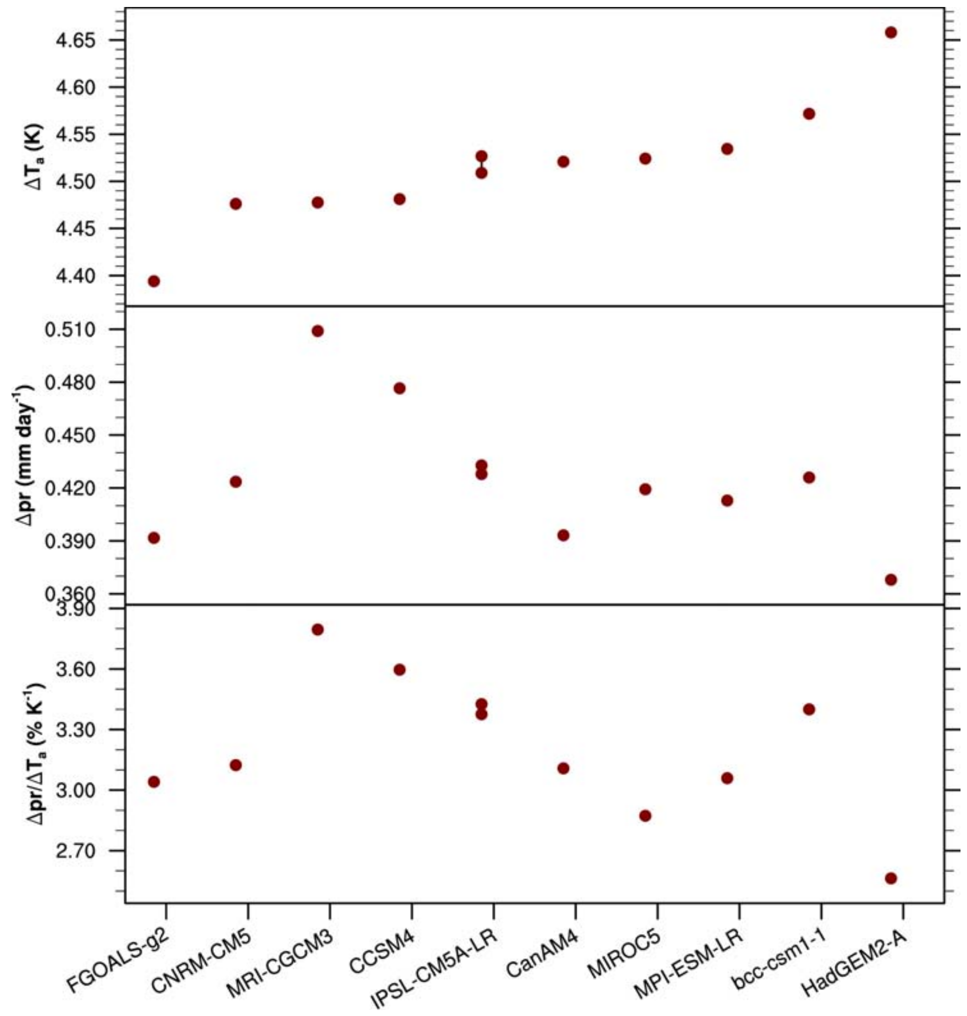

fig. S1. Hydrological sensitivity for fixed SST. Difference between the amip4K and the amip run in near surface air temperature (upper) and surface precipitation (middle) and the hydrological sensitivity computed from these differences (lower panel).

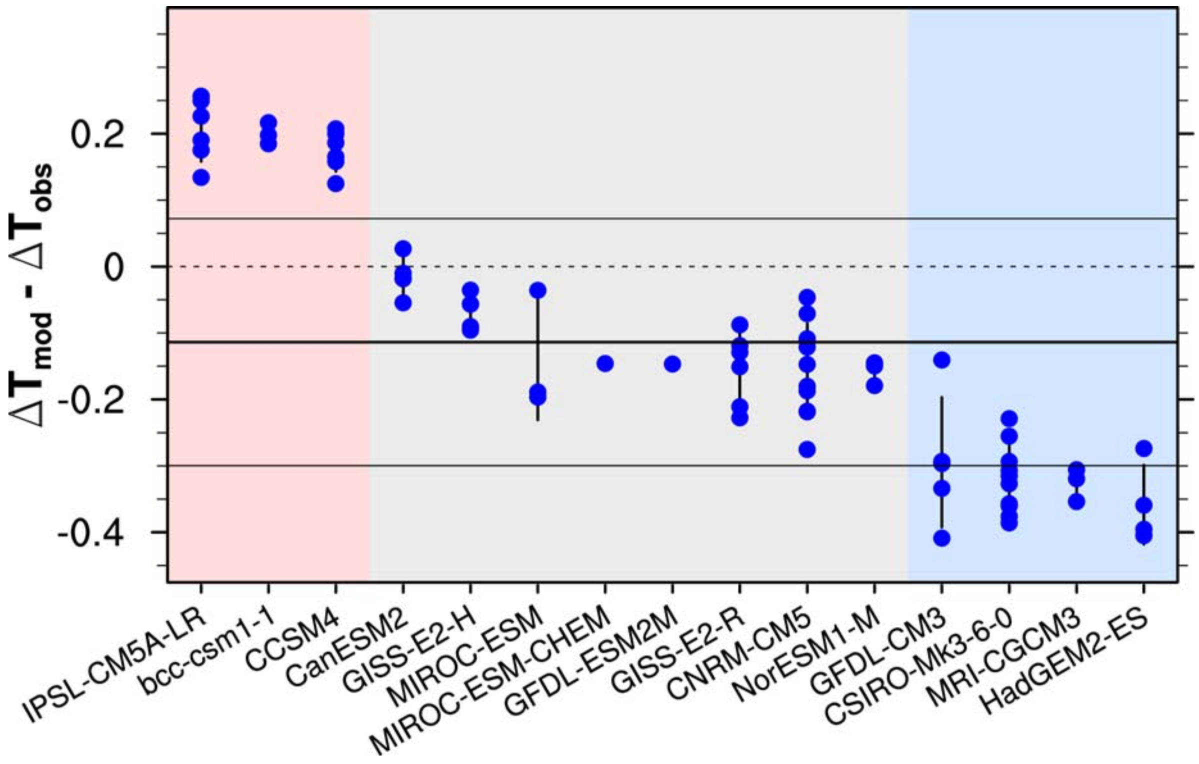

fig. S2. Grouping of models according to 20th century temperature increase. Difference ΔT_{mod}-ΔT_{obs} where ΔT_{mod} is the difference between the 1986-2005 and the 1901-1920 global average simulated surface temperature from the CMIP5 historical (all forcings) experiment. Here the surface temperature is defined as SST over ocean and near-surface air temperature over land. ΔT_{obs} is based on ERSST SST and CRU near-surface air temperature observation derived data. The shading indicates the grouping of the models into warm, medium, and cold. Solid horizontal lines indicate the multi-model average ± one standard deviation. Areas covered by sea ice have been masked out.

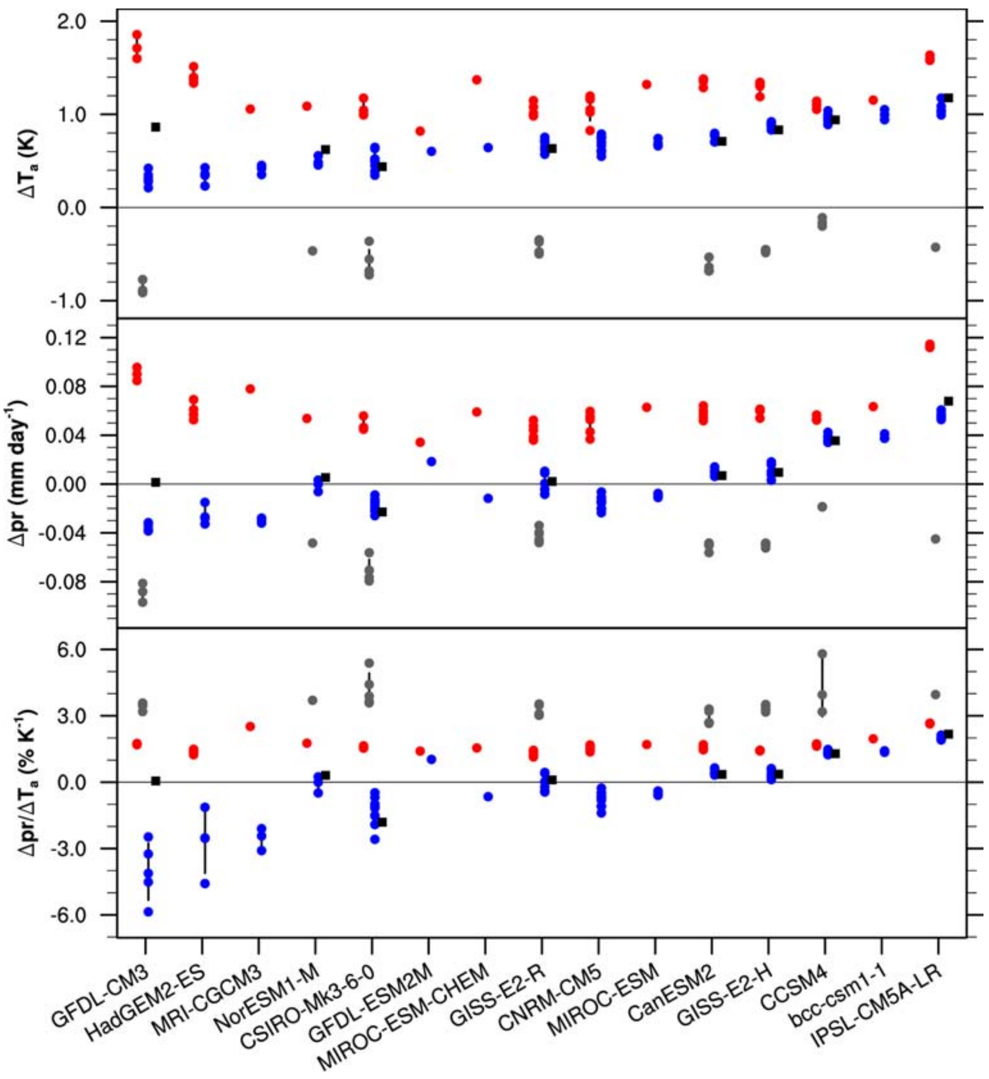

fig. S3. Response to GHG, aerosol, and all forcings from individual models. Difference between first and last 20 years from climate model runs with only GHG (red), only aerosol (gray), and all (blue) forcing for global mean near surface air temperature (upper), precipitation (middle), and hydrological sensitivity (lower panel) for various climate models. In the two upper panels, black squares are sums of GHG and aerosol only averages. In the lowermost panel, the black squares indicate the hydrological sensitivity computed with Equation 1. The runs started in 1850 (except a few that started in 1860) and ended in 2005.

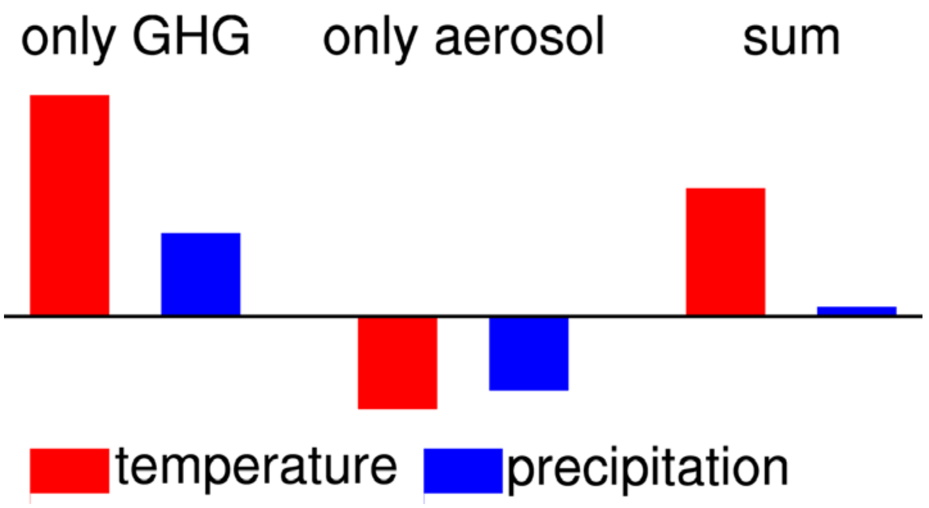

fig. S4. Schematic representation of the hydrological sensitivity to various forcings. Based on the averages of the historical sensitivity runs (for “medium” models).

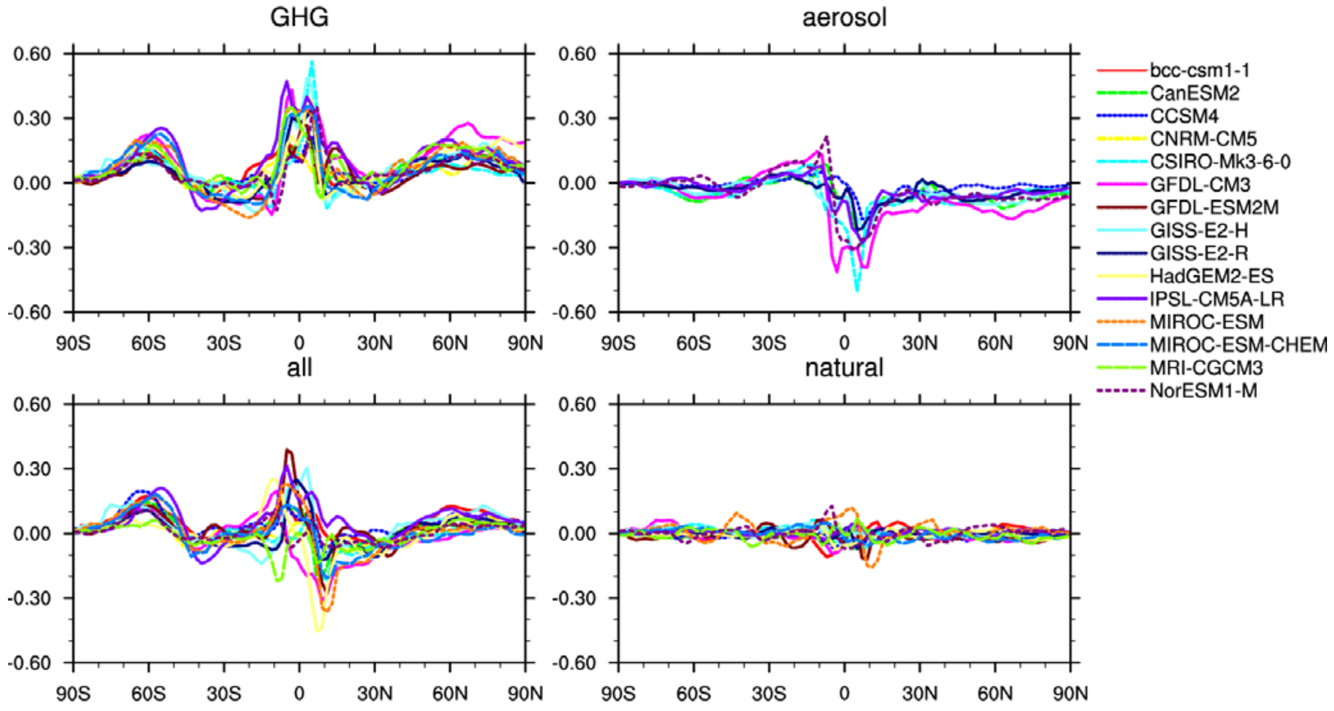

fig. S5. Zonal mean precipitation change from individual models. Difference of zonal mean surface precipitation (mm day^{-1} between the first and the last 20 years of the historical experiment with various forcings.

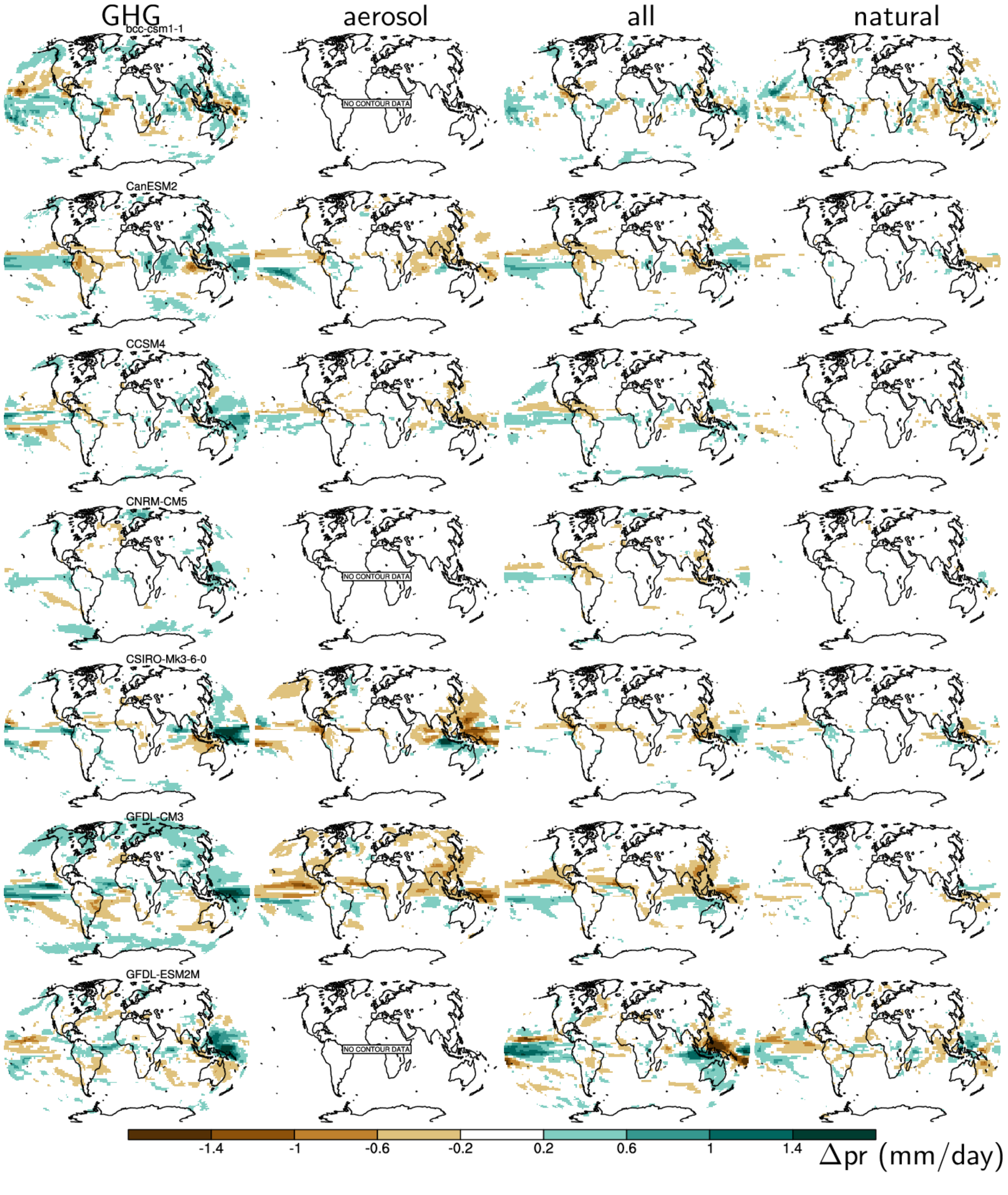

fig. S6. Maps of surface precipitation change from individual models (part1). Difference of surface precipitation (mm day^{-1}) between the first and the last 20 years of the historical experiment with various forcings.

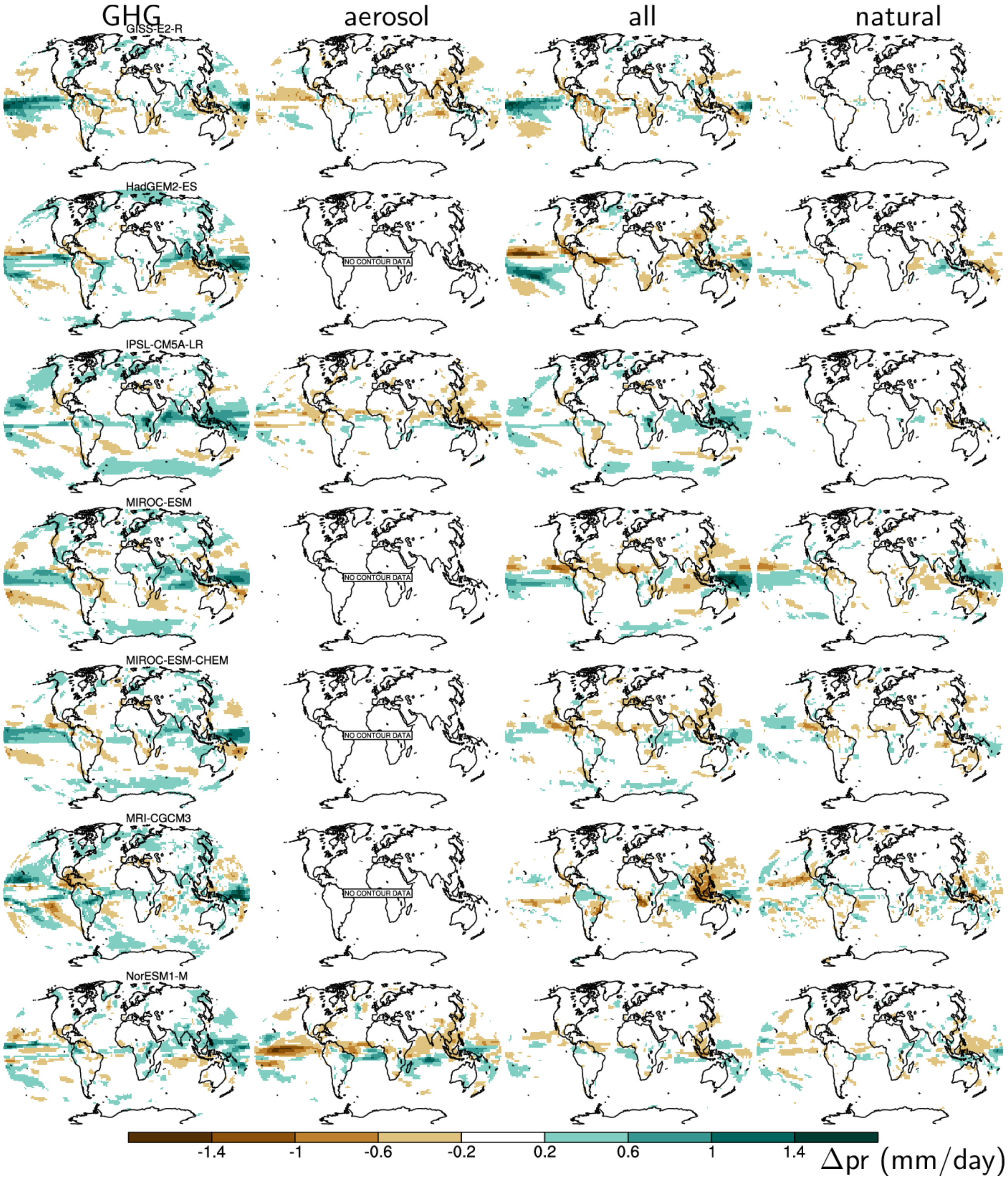

fig. S7. Maps of surface precipitation change from individual models (part2). Continued from fig. S6.

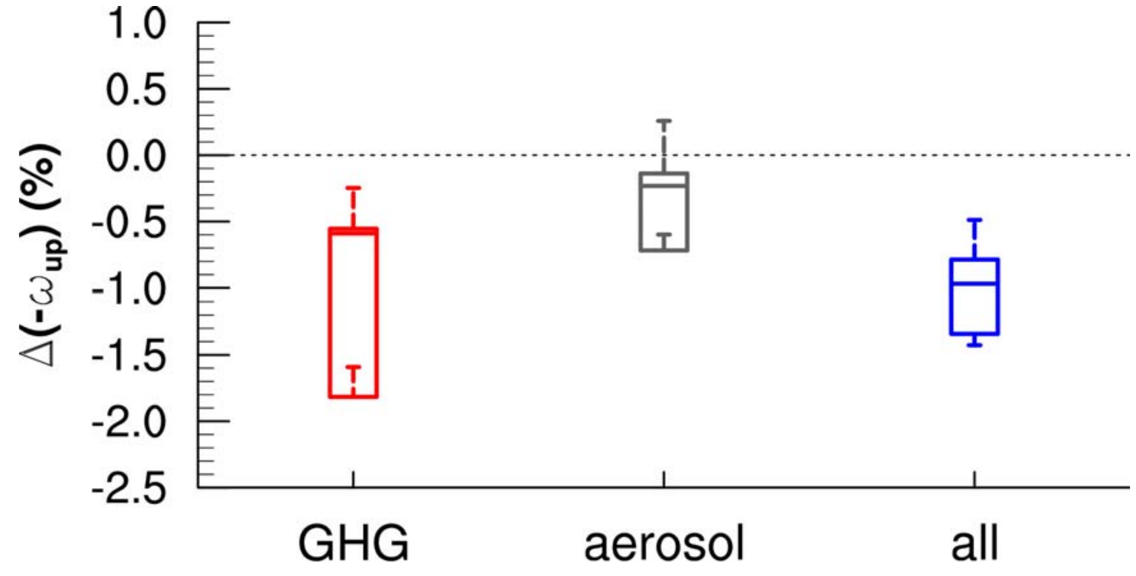

fig. S8. Global mean atmospheric overturning circulation changes for GHG, aerosol, and all forcings. Percentage difference of the multi-model mean negative of the global mean 850 hPa pressure vertical velocity in regions with upward motion $-\omega_{up}$ between the first and the last 20 years of the historical experiment for various forcings. Based on monthly mean data. Negative values indicate a weakened circulation.

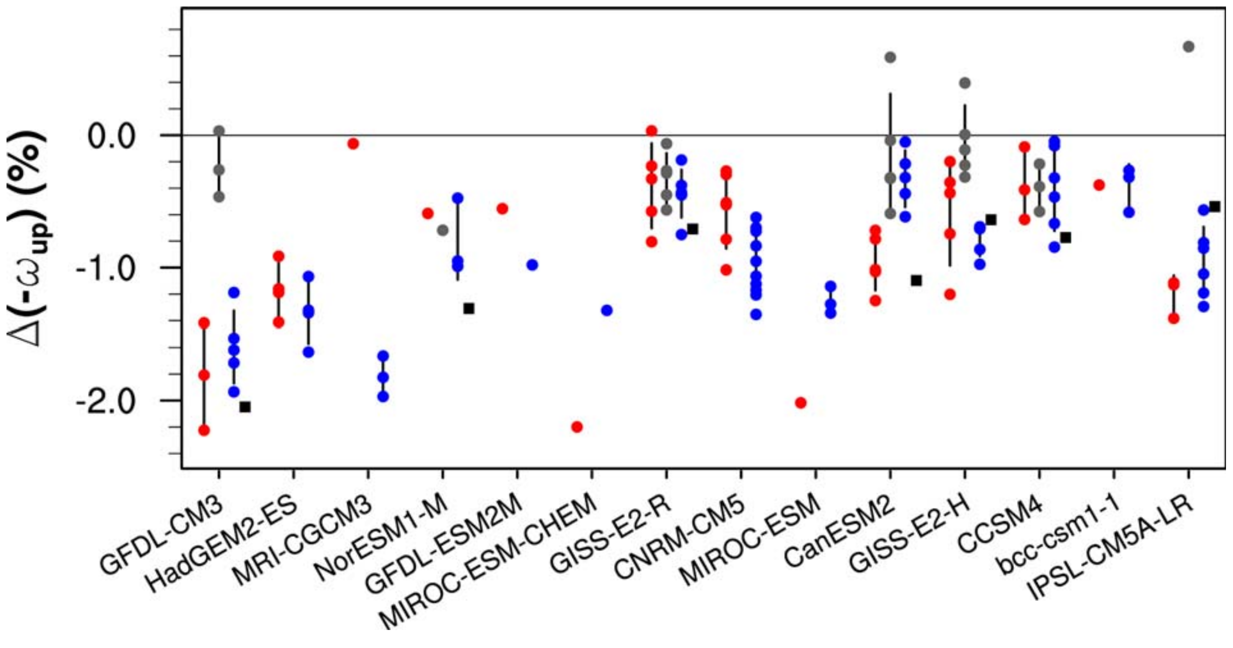

fig. S9. As fig. S8 for individual model runs. Symbols and colors as in fig. S3.

table S1. Hydrological sensitivity (% K^{-1}).

	cold	medium	warm	all
GHG	1.63 ± 0.30	1.48 ± 0.14	2.14 ± 0.49	1.72 ± 0.39
aerosol	3.89 ± 0.70	3.20 ± 0.32	4.22 ± 1.11	3.63 ± 0.49
all	-2.34 ± 1.43	-0.14 ± 0.59	1.61 ± 0.34	-0.40 ± 1.68
rcp45	2.31 ± 0.34	1.85 ± 0.23	2.10 ± 0.31	2.09 ± 0.36
rcp85	1.85 ± 0.35	1.52 ± 0.22	1.82 ± 0.28	1.67 ± 0.37
amip4K-amip	n/a	n/a	n/a	3.20 ± 0.36*

*result based on different set of models

table S2. Treatment of indirect (cloud-aerosol) radiative effects in historical runs. Includes only models from which the historical runs are used (i.e. models for which at least one single forcing run is available).

	albedo	lifetime
bcc-csm1-1	no	no
CanESM2	yes	no
CCSM4	no	no
GFDL-CM3	yes	yes
GFDL-ESM2	no	no
CNRM-CM5	yes	no
CSIRO-Mk3-6-0	yes	yes
GISS-E2-H	yes	no
GISS-E2-R	yes	no
HadGEM2-ES	yes	yes
IPSL-CM5	yes	no
MIROC-ESM	yes	yes
MIROC-ESM-CHEM	yes	yes
MRI-CGCM3	yes	yes
NorESM1-M	yes	yes

table S3. CMIP5 experiments used in this study.

CMIP5 experiment name	forcing	abbreviation
historicalGHG*	anthropogenic greenhouse gases	GHG
historicalMisc†	anthropogenic aerosol	aerosol
historicalNat	natural forcings, mainly volcanic and solar	nat
historical	GHGs, aerosol, and natural forcings	all
amip‡	prescribed SSTs, anthropogenic and natural forcings	amip
amip4K	same as amip, but SST increased by 4K everywhere	amip4K

*all historical runs span the years from 1850 to 2005 except for three models for which the historical runs start in 1960. The first and the last 20 years are analyzed.

†only sensitivity runs for anthropogenic aerosol

‡years 1979–2005

table S4. Number of runs per model used in this study.

**Model/forcing**	**GHG**	**aerosol**	**all**	**nat**	**rcp45**	**rcp85**	**amip**	**amip4K**
bcc-csm1-1	1	-	3	1	1	1	1	1
CanESM2/AM4	5	5	5	5	5	5	1	1
CCSM4	3	3	6	4	6	6	1	1
CNRM-CM5	6	-	10	6	1	5	1	1
CSIRO-Mk3-6-0	5	5	10	5	10	10	-	-
FGOALS-g2	-	-	-	-	-	-	1	1
GFDL-CM3	3	3	5	3	1	1	-	-
GFDL-ESM2M	1	-	1	1	1	1	-	-
GISS-E2-H	5	5	5	5	5	1	-	-
GISS-E2-R	6	5	5	5	6	1	1	1
HadGEM2-ES/A	4	-	4	4	4	4	1	1
IPSL-CM5A-LR	3	1	6	3	4	4	2	2
MIROC-ESM	1	-	3	1	1	1	-	-
MIROC-ESM-CHEM	1	-	1	1	1	1	-	-
MIROC5	-	-	-	-	-	-	1	1
MRI-CGCM3	1	-	3	1	1	1	1	1
MPI-ESM-LR	-	-	-	-	-	-	1	1
NorESM1-M	1	1	3	1	1	1	-	-
**total**	**46**	**28**	**71**	**46**	**48**	**43**	**12**	**12**
